# Hierarchical Porous Egg White Hydrogel Promotes Diabetic Wound Closure through Topography-Guided Cell Recruitment

**DOI:** 10.34133/bmr.0279

**Published:** 2026-02-11

**Authors:** Ting Su, Xingtang Niu, Xinhui Wang, Dan Sun, Mimi Xu, Yu He, Xiaoqi Huang, Yuan Ma, Qiang Chang, Feng Lu

**Affiliations:** Department of Plastic and Reconstructive Surgery, Nanfang Hospital, Southern Medical University, Guangzhou 510515, China.

## Abstract

Skin healing often results in scarring or pathological conditions like keloids due to abnormal cell proliferation. These outcomes are attributed to abnormal proliferation or functional defects in skin cells. Hydrogels, mimicking the extracellular matrix, can guide hierarchical cell alignment for improved regeneration. Inspired by egg white’s foaming ability, we engineered a bilayer hydrogel dressing: a porous dermis layer via whipped egg white and a dense epidermis layer crosslinked with calcium. The artificial egg white skin (EWS) was tested in in vitro cell culture and in vivo application on mouse wounds. RNA sequencing explored the specific mechanism of EWS on cells. EWS features a multistage macroporous structure mimicking skin’s longitudinal mechanical performance. This migration inducive property of egg white facilitates directional migration and allows for the vertical stacking of keratinocytes and fibroblasts. The collaboration of cells enhances expression of positive chemokines and growth factors, shortening inflammation reaction and improving wound healing. Transcriptome sequencing reveals a substantial up-regulation of genes related to cell cycle and metabolism. EWS offers a cost-effective and efficient platform for biomimetic skin dressing and shows potential for other applications in regenerative medicine.

## Introduction

Full-thickness skin defects caused by ulcers, chronic infections, and tumor excisions have been a troublesome problem that affect tens of millions of people worldwide [1]. The reconstruction of topical skin for the canonical acute traumatic defects is an intricate procedure that involves 4 temporally and spatially overlapping phases, namely, hemostasis, inflammation, proliferative, and remodeling phases [2]. Excluding superficial injuries, most skin defects often result in hypertrophic scars and keloids. Even if the wound is closed, the regenerative skin may still present cosmetic problems, functional contractures, or subjective symptoms such as pruritus and pain [3]. Uncontrollable hyperplasia of collagen fibers, persistent inflammation, and impaired cell function may be responsible for defective skin regeneration. The pursuit of novel materials for diabetic foot ulcer (DFU) healing extends beyond scientific curiosity; it is fundamentally driven by urgent, unmet clinical needs [4]. DFUs carry a devastatingly high risk of severe secondary complications, including life-threatening infections and limb amputations. Critically, dysfunctional immune cell responses—such as impaired macrophage polarization and persistent neutrophil activation—within the chronic wound microenvironment are a primary driver of these complications [5]. While numerous therapies target immune cells in wound healing, the ultimate objective remains promoting functional recovery of parenchymal skin cells—such as epidermal keratinocytes (Kc) and dermal fibroblasts (Fb). Hence, there is a compelling imperative to develop advanced therapeutic strategies, particularly biomaterials engineered to actively modulate aberrant immune activity and achieve clinically meaningful functional healing.

Novel paradigms of wound dressings based on biomaterials have emerged, opening exciting opportunities for wound healing nowadays. Instead of conventional gauzes that only provide simple functions of gas exchange and exudate absorption [6], biomedical hydrogels are believed to be a promising alternative for accelerating natural healing. Beyond acting as contamination barriers, moisture maintainers, infection preventers, and bioactive carriers, they mimic matrices promoting stromal cell development and tissue organization. Research focuses on biomimetic hydrogels with interfacial, chemical, mechanical, and biological functionalities like natural tissue [7]. Human skin comprises 2 interdependent layers: epidermis and dermis [1]. The epidermis is a densely packed layer composed of Kc and melanocytes, exhibiting a high degree of organization. The dermis has a more relaxed structure, containing Fb, immunocytes, and interstitial collagen fibers [8]. Many hydrogels have been developed for the purpose of skin regeneration, aiming to imitate extracellular components, create a suitable cell niche, and facilitate tissue growth. Wang et al. [9] have designed a bilayer sheets using silk fibroin and sodium alginate to mimic the hierarchy structure of skin, which showed apparent accelerated wound closure. Moreover, they developed an injectable extracellular matrix (ECM)-mimicking hydrogel (HOG@P&D) to achieve smart drug delivery [10].

Hierarchical structures can be observed across various scales, ranging from individual cells to the complex human body. Uniform pore size is inadequate for accurately replicating the intricate nature of the protogenic skin. Hierarchical porous materials, commonly referred to as mesoporous materials, exhibit a distinctive feature of possessing pore sizes that span multiple length scales [11]. Compared to simple porous materials, they offer high surface areas, large accessible space, controllable morphologies, and adjustable functions. For example, calcinating diatomite with surface-distributed nano-silica achieved rapid hemostasis via nano-to-micro pores, leading to the attainment of expedited hemostasis [12]. The incorporation of mesoporous bioactive glasses as implants has been advantageous due to their ability to strongly bind to bone, minimize diffusion barriers, facilitate drug delivery, and promote osteogenesis, angiogenesis, and anticancer capacity [13]. Similarly, bilayer hydrogels are developed for bionic dressings focusing on hierarchical porosity [14]. Current methods for producing hierarchical pores include templating methods (such as dual surfactant templating, emulsion templating, colloidal crystal templating, and polymer templating) and template-free methods (including spontaneous formation, sol–gel controlling, selective leaching, zeolitization, and post-treatment) [12]. However, both approaches necessitate the use of tailored molds, pore-foaming agents (some of which may possess biotoxicity), or intricate procedures [15]. Therefore, bio-friendly hierarchical hydrogels with feasible fabrication are needed.

Natural polymers, such as proteins, polysaccharides, and polynucleotides, exhibit a higher level of bioactivity and biodegradability compared to synthetic polymers in the field of regenerative medicine [16]. Collagen-based dressings like Fibrocol, a bovine-derived collagen film, are valued for their biocompatibility, yet they face challenges such as rapid enzymatic degradation, potential immunogenicity, and insufficient mechanical stability in dynamic wound environments [17]. Alginate dressings such as Kaltostat, derived from seaweed, excel in exudate absorption and moisture retention, making them suitable for highly exudative wounds. The inherent biocompatibility, non-immunogenicity, and cost-effectiveness of alginate dressings further enhance their clinical utility in wound management [18]. However, their poor mechanical stability at swollen state restricts their applicability across diverse clinical scenarios [19].

Egg white (EW) is an underestimated material with versatility, low cost, tunability (gelling, foaming, emulsifying, and bonding), and abundant biological elements (like lysozyme and ovotransferrin). The application of EW expands from being additives of hydrogel matrices to the main component of hydrogels. EWs are known to possess a diverse range of high-quality proteins that contribute to the formation of a voluminous foam and a well-structured macroporosity when subjected to whipping or beating [20]. Nevertheless, the pore structure achieved through this method is notably delicate, as collapse and phase separation typically occur within a duration of 30 min [21]. Studies have attempted to improve the porous structure of these sponges through various methods such as lyophilization, foaming of ovomucin, and the addition of gelatin–methyl anhydride, alginate, and poly(ethylene oxide) [22]. However, the resulting pore sizes consistently fell within a unimodal range or failed to maintain the desired macroporosity. Additionally, in the preparation of pickled eggs, mud, and plant ash, metal cations (K^+^, Ca^2+^, Zn^2+^, and Mg^2+^) are commonly employed to enhance the protein’s stability [23]. In a similar manner, the inclusion of cations was employed subsequent to the initial gelation process in order to establish additional ionic bonding between the protein molecules [24]. Consequently, the introduction of metal ions through a secondary cross-linking process offers the potential to adjust pore sizes and enhance mechanical properties. Our prior work confirmed that EW hydrogels promote diabetic wound healing via intrinsic bioactivity without added growth factors [25]. EW hydrogels have been found to possess certain characteristics, including the ability to promote proliferation, vascularization, and diabetic wound healing, without the need for additional growth factors, as observed in other hydrogels.

Herein, we fabricate hierarchically porous materials mimicking skin structure, using EW’s foaming and gelation. Primary whipping and alkali-induced gelation were found to generate micropores and macropores simultaneously. A gradient cross-linking process with divalent cations formed a tightly packed epidermis layer that seamlessly interlocked with a loosely structured dermis layer, creating a cohesive mimetic skin dressing. The mechanical variations at different layers create customized environments for cells and promote early communication in in vitro coculture, eliminating the need for additives. The hierarchical egg white skin (EWS) has potential for use in skin regeneration and other medical applications due to its low cost, ease of synthesis, and adjustable mechanical properties.

## Materials and Methods

### Sequential synthesis of hydrogels

EW was carefully separated and whipped to foam by an electric stirrer at 3,000 rpm in 45 s. EW foam was fully mixed with 1:1 NaOH (0.5 M) to form the foam hydrogels. The hydrogels were tailored into cylinders (10 mm in diameter and 5 mm in height). The cylinders would be called EW macroporous (EWM) hydrogels if without other treatment, and EWM-Ca hydrogels were those completely crosslinked with 2 M CaCl_2_. If only the underside of the cylinders is allowed to contact with a CaCl_2_ solution, a reaction with calcium ions will form an epidermal layer, leading to the formation of EWS hydrogels. All the hydrogels were sterilized with 75% ethanol overnight and completely rinsed with phosphate-buffered saline (PBS) buffer.

### Physical characterization of hydrogels

#### SEM

The microstructures of the hydrogel were analyzed with a scanning electron microscope (ZEISS GeminiSEM 500, Germany). Briefly, the samples were freeze-dried (Christ Alpha 2–4 LD Freeze Dryer) for 24 h and then sprayed with gold (20 nm, Edwards sputter coater) to observe under an operating voltage at 5 kV.

#### FTIR

The raw EW, EW foam, and EWS were lyophilized for FTIR test. FTIR spectra of 3 kinds of samples were recorded by the Nicolet iS50 FTIR spectrometer equipped with attenuated total reflection module with a wavelength range from 4,000 to 400 cm^−1^ along with a 1 cm^−1^ resolution.

#### Rheology test

Dynamic rheology experiments were performed using a HAAKE MARS 40 photo-rheometer with parallel-plate (P20 TiL, 20 mm diameter) geometry at 20 °C. Time-sweep oscillatory tests were performed at a 10% strain, 1-Hz frequency, and a 0.5-mm gap (CD mode) for 60 s, and the gel point was determined at the time when the storage modulus (*G*′) surpassed the loss modulus (*G*″). Oscillatory frequency sweep tests were performed over a frequency range of 0.1 to 100 rad/s at a constant strain amplitude of 1%. The shear-dependent viscosity performance was monitored with the shear rates ranging from 0.01 to 1 s^−1^.

#### Compression test

The compressive stress–strain measurements were performed using a tensile-compressive tester (INSTRON Model 5567 machine with a 50-N sensor). The samples were formed into a cylinder with a height of 10 mm and a diameter of 20 mm. The compressive strain rate was 5 mm/min, and all tests were repeated 3 times for each group. Stain was limited to 80% as constructed. The correlation curve was drawn with strain (ε) as the abscissa and stress (σ) as the ordinate. The compressive moduli were the approximate linear fitting values of the stress–strain curves in the strain range of 10% to 20%. Equation (1) was used to calculate Young’s modulus (*E*):

E=σ/ε (1)

#### Air permeability test

The air permeability was evaluated through the Air Permeability Tester MO21A (SDL ATLAS). Hydrogels( 20 cm^2^) were pressed on the testing head of the instrument. The test was performed at 100-Pa pressure and repeated for 8 times to obtain the mean values.

#### Moisture vapor transmission rate

The moisture vapor transmission rate (MVTR) was determined using a standardized gravimetric method. The hydrogels were sandwiched between 2 silicone gaskets and mounted in Paddington Cups (2 cm diameter). Ten milliliters of deionized water was added into the cup. The air gap between the liquid surface and the hydrogel was then controlled to be 5 mm. The whole instrument was measured and placed at 37 °C for 24 h (Δ*t* = 1), and then measured again to obtain the mass difference (Δ*m*). Equation (2) was used to calculate MVTR, in which *A* means area:

$$\text{MVTR}=\frac{\Delta m\times \Delta t}{A}$$ (2)

#### Water retention rate

A solution containing 0.368 g of anhydrous calcium chloride, 2.298 g of sodium chloride, and 1 l of deionized water was prepared and prewarmed to 37 °C, after which the hydrogels were cut into 2 cm × 2 cm × 1 cm cylindrical specimens, weighed, and placed in culture dishes into a 37 °C constant-temperature incubator; a solution volume equivalent to 100 times the sample mass was added. Samples were retrieved every 6 h, suspended for 30 s to remove excess fluid, and reweighed—this procedure was repeated for 5 independent trials. The water absorption capacity was calculated using Eq. (3), where *m*_t_ represents the post-absorption mass and *m*₀ denotes the initial mass:

$$\text{Water rentention rate}=\frac{\left(m_{t}-m_{0}\right)\times 100\%}{m_{0}}$$ (3)

### Cell isolation

Primary mouse Kc were isolated from the dorsal skin of newborn C57BL/6 mice and cultured as the reported method [26]. Briefly, the separated skin was placed on a petri dish, dermis-side down, and flattened. After incubating the skin floated on 0.25% trypsin (Gibco; Thermo Fisher, Waltham, USA) at 4 °C for 18 h, we transferred the skin to a new petri dish, epidermis-side down, and carefully removed the dermis with forceps. The epidermal sheets were pooled with specific reduced calcium medium and then minced into pieces. The cell suspension was centrifuged, and the sediment was resuspended and filtered through a 100-μm cell strainer. After recentrifugation, Kc were isolated and cultured in the plates. Another dorsal skin was harvested and cut into pieces to incubate with the mixture of collagenase I (Gibco) and Dulbecco’s modified Eagle’s medium (DMEM) containing 10% fetal calf serum (Gibco) at 37 °C for 6 h. The cell suspension was centrifuged, and the sediment was resuspended and filtered through a 100-μm cell strainer. Then, Fb were cultured in the plates. Passages 3 to 5 of these 2 cells were used in this research.

### In vitro cell experiment

For cell tracing on EWS hydrogels, according to the manufacturer’s instruction, Fb and Kc were labeled with DiO and DiI (Cell Plasma Membrane Staining Kit, Beyotime, Zhengzhou, China), respectively. Kc was seeded on the EWM-Ca hydrogels at a density of 1 × 10^5^ cells. Fb was seeded on the EWM hydrogels at a density of 5 × 10^5^ cells. For bilayered coculture, culture dishes were precovered with agarose. Then, the upper surface of EWS was immersed in Kc suspension for 24-h incubation. Next, the EWS was overturned to make the bottom to contact the Fb cell suspension for 4 d. Fluorescent images were photographed with an inverted fluorescence microscope (Ti2, Nikon, Japan). Three-dimensional (3D) images were reconstructed with a laser scanning confocal microscope (LSM980, Leica, Frankfurt, Germany).

For cell viability measurement, the leaching solution of EWM or EWM-Ca was added in the culture medium at a volume ratio of 5%. Fresh EW fluid (5%) was added to the medium of the EW group as another control. After specific culture time, cells underwent 3-time washing and incubated with 2 μM calcein and 4 μM ethidium homodimer-1 solution (Viability/Cytotoxicity Kit, Thermo Fisher, Waltham, USA) for 30 min. Live cells showed green and dead cells stained red when observed with a fluorescence microscope (Ti2, Nikon). For proliferation assessment, Cell Counting Kit-8 (CCK-8) (Solarbio) was used under standard protocols of the manufacturer’s instruction. optical density (OD) values of cells were measured with a multimode reader (Varioskan LUX, Thermo Fisher)

A modified scratch assay was conducted with the assistance of transwell plates. Cells were cultured on the lower chambers until cells reach 90% confluence. The cell layer was scraped in a straight line using a 1-mm pipette tip and then gently washed with PBS to remove detached cells. Then, hydrogels were placed on upper chambers. The same spot was observed with a microscope, and migrated area was measured using Fiji (v1.54).

### In vivo wound healing experiments

Full-thickness skin wounds were performed to reveal the wound healing properties of EWS. All animal procedures were approved by the Ethics Committee of Southern Medical University (approval no. IACUC-LAC-20250612-001) and strictly followed the National Institutes of Health (NIH) Guide for the Care and Use of Laboratory Animals. C57BL/6 mice (4 to 6 weeks) or db/db mice (4 to 6 weeks) were anesthetized by sevoflurane via inhalation, and the dorsal surface was shaved and sterilized with povidone iodine. The wound model was made by slicing a circular full-thickness defect 10 mm in diameter centered over the spine. A silicone ring splint with the same inner diameter was anchored beyond the wound periphery to avoid skin contraction. Eighteen mice were divided randomly into 3 groups as designed: empty control (CON), EWM hydrogels (EWM), and EWS hydrogels with Fb and Kc cells (EWS). Each mouse was marked on non-wound-area skin using a gentian violet skin marker. All the wound beds were covered by commercially available 3M transparent film dressing. Afterward, the animals were kept in separate cages, the wound photographs were taken after the designed days, and the wound area was analyzed by ImageJ software. The wound area % during time was measured by comparing it with the original wound area.

### Section staining

After the animals were sacrificed on the designed days, the wound area with peripheral normal skin was surgically removed. The samples were fixed in formaldehyde solution, dehydrated, embedded with paraffin, and cut into 4-μm sections.

For hematoxylin and eosin (H&E) staining, sections were deparaffinized in xylene (2 × 10 min), rehydrated through ethanol series to water, stained in Harris hematoxylin (5 min), differentiated in 0.5% HCl/70% ethanol (5 s), blued in ammonia water (5 s), counterstained in eosin Y (3 min), dehydrated through graded alcohols, cleared in xylene, and mounted with resinous medium.

For Masson’s trichrome staining, sections were deparaffinized and hydrated to water. Nuclei are stained with Weigert’s iron hematoxylin (10 min), washed under tap water (5 min), briefly differentiated in 1% HCl/70% ethanol (10 s), and blued in alkaline water. Sections are then stained in Biebrich scarlet-acid fuchsin (5 min) for cytoplasm/muscle, differentiated in phosphomolybdic acid (10 min), counterstained with aniline blue (5 min) for collagen, and rinsed in 1% acetic acid (1 min). Then, the sections went through dehydration with graded alcohols, clearance with xylene, and mounting with resinous medium.

For immunofluorescence analysis, the processed sections were boiled in citrate solution and hydrogen peroxide (3%) and underwent blocking in goat serum (2.5%). Overnight incubation with varying primary antibodies was conducted, including anti-TNFα (tumor necrosis factor α) monoclonal antibody (Abcam, ab183218, 1:2,000), anti-αSMA (α-smooth muscle actin) antibody (Abcam, ab184705, 1:200), anti-vimentin monoclonal antibody (Abcam, ab92547, 1:500), anti-CD31 antibody (Abcam, ab182981,1:2,000), and anti-F4/80 antibody (Abcam, ab300421, 1:500). After thorough washing with buffer and incubation with secondary fluorescent antibody (Abcam, ab150077 and ab150080,1:200) and 4′,6-diamidino-2-phenylindole (DAPI) (Solarbio), the samples were observed with a confocal microscope and analyzed with ImageJ.

### RT-qPCR analysis

Control groups of Kc and Fb were kept in adherent culture, while the EWS group was cultured as mentioned above. In vitro samples were collected on the 5th day via the Total RNA Extraction Kit (Solarbio). After RNA extraction, RNA samples were then reverse-transcribed to cDNA using Revert Aid First Strand cDNA Synthesis Kit (Thermo Scientific). Then, LightCycler quantitative polymerase chain reaction (qPCR) instrument (Roche, Indianapolis, IN, USA) and SYBR Green Master Mix (Beyotime) were employed for reverse transcription PCR (RT-PCR) measurement according to the manufacturer’s instruction, the Gene expression was calculated as Ct, and relative expression levels were calculated with the 2^−ΔΔCt^ method. The PCR primers are listed in Table S1.

### RNA sequencing

EWM hydrogels were prepared in a 10-cm culture dish, soaked in PBS after alcohol disinfection, and thoroughly rinsed. The experimental group (M) was set up, while the control group (C) was empty. L929 cells were seeded at a concentration of 2 × 10^6^ cells, with 3 replicates for each group. The medium was changed the following day, and after 7 d of cultivation, the culture medium was discarded. PBS was then added for 2 washes. Trizol lysis solution was added to obtain total RNA. The extracted RNA was stored in liquid nitrogen and sent for testing. After the test, library construction was performed, followed by high-throughput sequencing using the Illumina HiSeq platform. The raw sequencing data were filtered and aligned to the reference sequence (Hisat2). Gene quantification analysis was performed based on the alignment results. Gene expression levels in different sample groups were analyzed for correlation and differential expression. The fold change (FC) represents the ratio of expression levels between 2 samples. The screening criteria were set as |log_2_FC| > 2 and *P*_adj_ < 0.01 after multiple testing correction. The differentially expressed genes (DEGs) that were identified underwent various analyses, including Gene Ontology (GO) functional analysis, Kyoto Encyclopedia of Genes and Genomes (KEGG) analysis, clustering analysis, and protein interaction network mining. These analyses aimed to uncover the impact of EWS on cell function and metabolic pathways.

### Transmission electron microscope of wound samples

Diabetic mice were shaved and randomly assigned to 2 groups and received full-thickness wound surgery. On 14th day post-surgery, the wounds were excised into approximately 1-mm^3^ pieces and immersed in 2.5% glutaraldehyde at 4 °C overnight, followed by a graded series of ethyl alcohol and acetone solutions for dehydration. The samples were subsequently embedded in an Epon–Araldite mixture, and tiny sections were prepared and stained with uranyl acetate and lead citrate. Micrographs were captured using an HT7800 transmission electron microscope (Hitachi, Japan) at magnifications of 1,000× and 3,000×.

### Statistical analyses

All data were presented as mean ± SD. Statistical differences between groups were analyzed by using one-way analysis of variance (ANOVA) with Tukey’s post hoc analysis test. Data are presented as means with 95% confidence interval. For all statistical tests, *P* < 0.05 was considered statistically significant.

## Results and Discussion

### Synthesis and characterization of EWS

Resembling the natural skin architecture as closely as possible is an important aspect of biomimetic hydrogel fabrication. The mechanical properties of human skin are anisotropic, viscoelastic, and nonlinear [27]. Typically, epidermis is the outermost layer of skin, undertaking the critical role as the defender of extraneous risks like mechanical and chemical strikes, heat, pathogens, and electromagnetic radiation as well as the regulator of body fluid evaporation [28]. It is supposed to be compact and elastic but porous to connect inner skin and outer air. Dermis is the organized, soft, uneven mesenchymal component formed by collagen and elastic fibers. It is highly interconnected for nourishment and innervation, supporting the growth of vessels, nerves, and multifarious appendages [29]. Therefore, dermis layer is relatively more porous than epidermis but with adequate mechanical strength to support the growth of Fb. To mimic the mechanical characteristics of natural skin, EW was used to fabricate the main framework based on its intrinsic foamability and physical cross-linking. EW consists of a variety of proteins, such as albumin, ovotransferrin, and ovomucoid. When subjected to a certain degree of unfolding and rearrangement, these proteins are rapidly absorbed at the air–liquid interface and polymerized into large-scale porous networks in the hydrogel [21]. Regarding the mechanism of alkali-induced gelation, EW proteins contain abundant thiol groups (-SH), and disulfide bonds are enhanced under alkaline conditions. This enhancement occurs via oxidation and SH–SS bond exchange reactions, which promote the formation of a strong hydrogel network. On the other hand, electrostatic repulsion also exerts effort in gelation [30]. In an alkaline environment, carboxyl (-COOH) was deprotonated to -COO^−^, and net charges on their surfaces forced denatured protein molecules to align and form a network structure [31]. Yet, the porous EW hydrogel manufactured in this way mimicked only the winding or bunching dermis fibers. To further modulate the epidermis-mimicry pore structure, secondary cross-linking by metal ions could be added. For the choice of a divalent cation, according to the report of Tian and his colleagues [32], calcium chloride (CaCl_2_) induced higher gel strength than other metal cations since Ca^2+^ is involved in the formation of bridge bonds between carboxyl groups. Therefore, we placed the hydrogels in shallow CaCl_2_ solution, leading to collapse and shrink of the contact area, which fabricated a condense layer on the macroporous EWM.

**Fig. 1.**
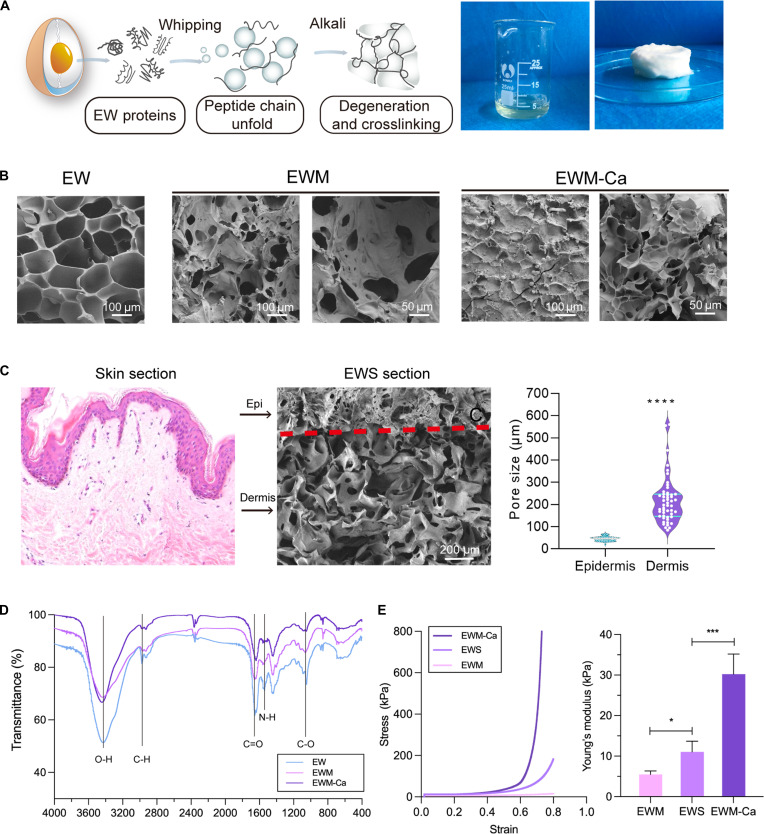
Production and physical characterization of EWS. (A) Sketch of EW foaming process and the gross picture of EWM. (B) SEM of the EW, EWM, and EWM-Ca. (C) Comparison of the layered structure of murine skin and EWS, and the pore size graph of different layers (*n* = 4). (D) Infrared spectrum and (E) Young’s modulus of EW, EWM, and EWM-Ca (*n* = 6). ***P < 0.001, ****P < 0.0001.

The gross and SEM images of intermediate and final products during fabrication are shown in Fig. 1. Through systematic optimization of whipping speed and duration, 3,000 rpm applied for 60 s was identified as the optimal condition (Fig. S1A and B), yielding consistent large pore sizes and solid physical strength (Fig. S1C to E). Whipping brought an interpenetrating structure with pores at the macroscale and microscale (Fig. 1A). *G*′ surpassed *G*″ within 5 s, indicating the rapid transformation from liquid to semisolid (Fig. S2A). Moreover, the newly formed hydrogel showed classical shear thinning behavior (Fig. S2B), which may facilitate the following 3D extrusion print (Fig. S2E). The cyclic rheological test and self-healing test reveal the structural stability of EW hydrogels under repetitive mechanical action (Fig. S2C and F).

Calcium was used for secondary cross-linking with EW proteins to make a macroscopically shrunken surface (Fig. 1B). The pore size of EWM-Ca hydrogels demonstrated a concentration-dependent reduction with increasing calcium ion concentration, while their elastic modulus exhibited a corresponding enhancement (Fig. S3A to C). The ultimate EWS hydrogels manifested a similar layered structure to mouse skin (Fig. 1C). The microscopic image of EWM-Ca demonstrated a dense appearance, while the macroporous interconnected network was successfully exhibited in EWM. Calculation of pore sizes in each layer also showed a significant difference, with EWM at 45.7 ± 10.6 μm and EWM-Ca at 214.1 ± 99.5 μm. In addition, there are super-large pores in EWM ranging from 400 to 600 μm. Although prior studies indicate that freeze-drying may induce macropore formation [33], our optical micrographs of fresh EWM confirm that these macroporous architectures (400 to 600 μm) predominantly arise from the foaming process rather than freeze-drying, as substantiated in Fig. S4C.

The infrared spectrum is used to investigate the molecular structure changes of EW produced by alkali and calcium (Fig. 1D). The C=O peak in the amide I region of EW at 1,657.1 cm^−1^ significantly decreases and shifts to 1,653.6 cm^−1^ after gel formation. Similarly, the peak in the amide II region at 1,538.9 cm^−1^ shifts to 1,541.8 cm^−1^, indicating a weakening of hydrogen bonding in the peptide bond C=O group [34]. The carboxyl group (-COO-) exhibits strong symmetric stretching peaks at 1,610 to 1,560 cm^−1^ and weak antisymmetric stretching peaks at 1,440 to 1,360 cm^−1^. The C–O absorption peak of EW at 1,403.9 cm^−1^ shifts to 1,400.0 cm^−1^ after gelation, and the intensity of the absorption peak increases significantly. The C–O stretching peak at 1,446.3 cm^−1^ shifts to 1,437.6 cm^−1^, indicating an increased deprotonation of -COOH in an alkaline environment [35].

After crosslinking with calcium, the absorption peak of -OH in EWM shifted from 3,425.0 cm^−1^ to 3,443.3 cm^−1^, and the peak intensity increased. This may be due to the formation of coordination bonds between nitrogen atoms and calcium ions, where the nitrogen atoms provide their electron pairs and hydrogen bonds are replaced by N–Ca bonds [36]. The C=O peak of amide I in EWM shifted from 1,653.6 cm^−1^ to 1,637.3 cm^−1^, while the N–H peak in amide II showed no significant change after calcium crosslinking. This suggests that C=O in the peptide bond may be involved in calcium chelation [37]. Overall, the main calcium binding sites on the serum macroporous hydrogel are carboxyl groups and peptide bonds. Acidic amino acids in the peptide sequence can serve as the primary calcium binding sites by providing carboxyl groups.

The stiffness of EWM hydrogels is supposed to meet the mechanical acquirement of skin tissue. Compression tests are used to compare the mechanical properties of 3 forms of macroporous hydrogels: EWM, EWM-Ca, and EWS (Fig. 1E). Within the 80% strain range, EWM has the lowest stiffness (5.49 ± 0.86 kPa), EWM-Ca has the highest stiffness (31.11 ± 8.67 kPa), and the stiffness of EWS is between the 2 (10.23 ± 8.91 kPa). Although EWS and EWM have the same main components, the hardness of EWS is significantly higher than that of the monomeric EWM (*P* = 0.025). This is attributed to the potential influence of the calcium-crosslinked epidermal layer. It is also closer to the stiffness range of the dermis layer of human skin (27 to 52 kPa) [29]. Chen et al. [38] found that Fb on gelatin methacrylated hydrogel (GelMA) with specific stiffness (10 to 40 kPa) demonstrated a more elongated morphology and significantly higher expression of fibrosis-related genes. Hydrogel stiffness also strongly influences the behavior and differentiation of mesenchymal stem cells (MSCs) and adipose-derived stem cells (ASCs) within them [39]. In this way, we obtained a biomimetic artificial skin hydrogel that mimics the morphology, structure, and stiffness of natural skin. This lays the foundation for subsequent in vitro cultivation of skin stem cells and the construction of artificial skin.

EWS hydrogels required excellent air permeability, moisture vapor transmission, and water retention capacity to address the moist and complex wound environment. The macroporous structure of the hydrogel facilitates the permeation of moisture vapor, while its robust mechanical strength contributes to maintaining the hydrogel’s water retention capacity. The EWS hydrogels exhibit an air permeability of 32 mm/s/cm^2^ (Fig. S2D), with an MVTR of 6,790 g/m^2^/day (Fig. S2E), which demonstrated naturally high breathability. Additionally, EWS can retain water up to 600% of its own weight without structural deformation (Fig. S2F), making it highly effective for managing heavily exuding wounds, while conventional GelMA hydrogels are more suitable for low exudate wounds.

### Biocompatibility and bioactivity of EWS

With abundant high-bioactivity components, EW and its derivatives were reported to show bacteriostatic activity, cell adhesivity, and growth factor binding [22]. The proteomic assessment of our previous study indicated that current physically crosslinked EW gelation preserved most functional proteins apart from membrane and extracellular region parts, whereas proteins promoted angiogenesis, cell adhesion, and migration [25]. Other than serving as hydrogels that fundamentally provide physical and spatial support, EWS was supposed to be conducive to cell functions and tissue regeneration as an engineered functional organoid.

**Fig. 2.**
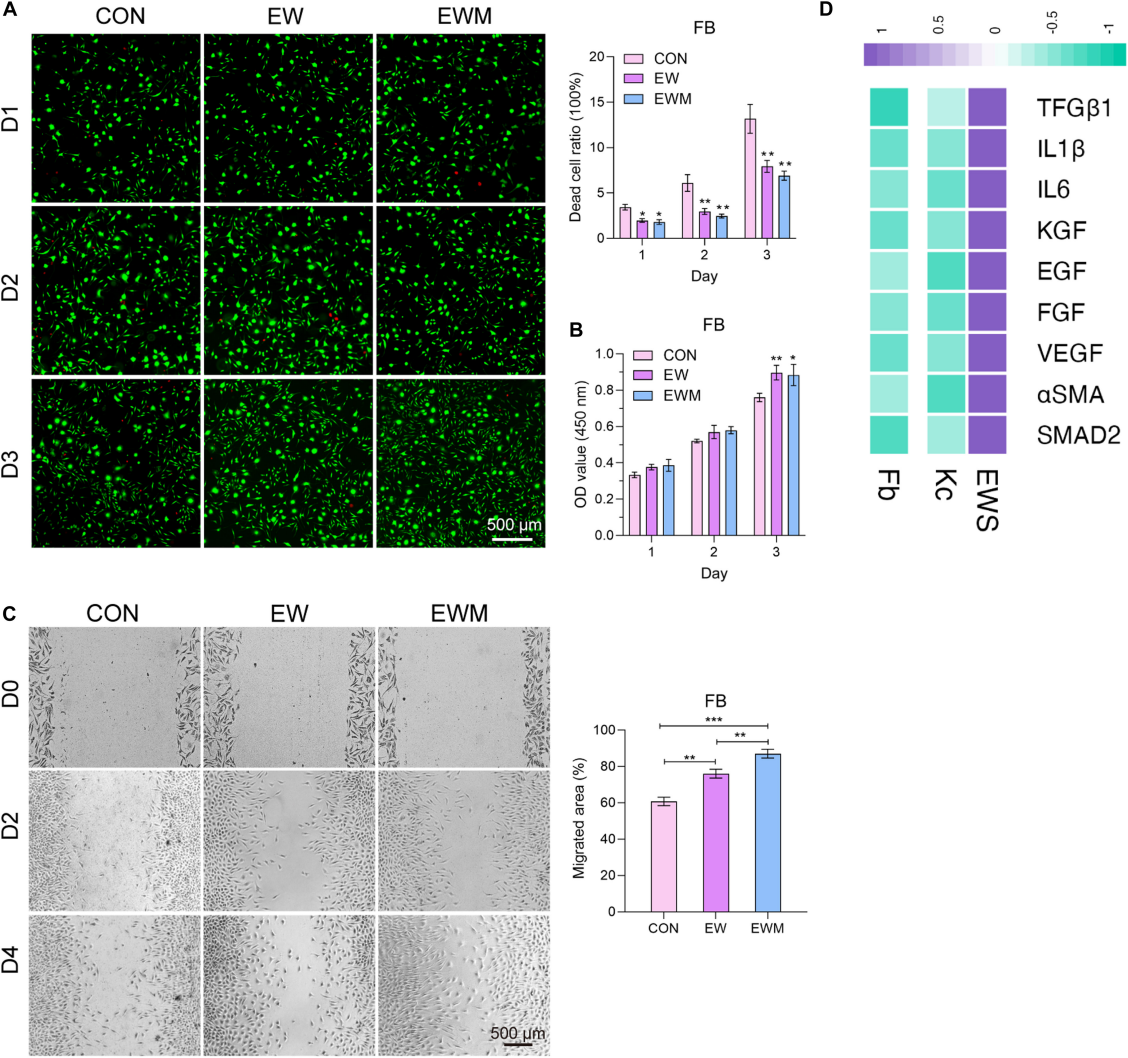
EWS served as a compatible hydrogel for cells. The scale bar applied to all panels. (A) LIVE&DEAD staining images and calculation of living Fb cells cultured on EWM (*n* = 4). (B) CCK-8 assay results showed the stimulative effect on cell proliferation (*n* = 4). (C) Scratch assay of Fb cells on EWM hydrogels (*n* = 4). (D) RNA expression of growth factors of cocultured cells on EWS (*n* = 4). *P < 0.05, **P < 0.01, ***P < 0.001.

After complete removal of residual OH^−^ and Ca^2+^ ions from the previous production process through immersion in DMEM (4 °C, 24 h), the leaching solution of EWM was added in the culture medium at a volume ratio of 5%. For EW control, fresh EW was added to the culture medium. Both EW and EWM exhibited significantly lower dead cell ratio compared with the control group, yet no difference was shown between the EW group and the EWM group (Fig. 2A and Fig. S4A). We continued to test the effect of hydrogel on cell proliferation activity. On the 3rd and 5th day, EW and EWM groups exhibited significantly higher OD values compared to the control (Fig. 2B and Fig. S4B). Previous studies have also found that serum hydrogels promote the proliferation of adipose stem cells, which may be attributed to the biological peptide function of serum [40]. The experimental results suggested that physical crosslinking does not affect the cell proliferation-promoting beneficial components in EW.

To explore the influence of EWM on cell migration, the modified scratch test was performed using transwell plates. The optical microscope images showed that EWM significantly promoted cell migration in the lower chamber (Fig. 2C), and EWM-Ca also plays a stimulative role on Kc cells (Fig. S4B). The EWM group revealed better promoting effect on cell migration than raw EW, indicating that foaming and physical gelating of EW help to release more potential components.

Intriguingly, the coculture system of Fb and Kc on EWS may somehow activate cross-talk between 2 cell types. The qPCR assay has been carried out to investigate the relative gene expression levels of Fb and Kc cells that were incubated on EWS for 5 d. It was astonishing to find that genes related to differentiation, growth, migration, and vascularization were greatly up-regulated compared to cells separately cultured on dishes (Fig. 2D). EWS provides an optimal environment for Fb and Kc to perform cell signal communication. The activated expression of multitudes of chemokines and growth factors may fertilize the wound bed and advance tissue regeneration. The hierarchical architecture of EWS, mimicking the epidermis and dermis layers of natural skin, provides tailored mechanical properties and pore size gradients. This design facilitates cell-specific microenvironments, enhancing Kc–Fb crosstalk and up-regulating critical growth factors [e.g., vascular endothelial growth factor (VEGF), transforming growth factor β (TGFβ), basic fibroblast growth factor (bFGF)], which collectively accelerate re-epithelialization, collagen remodeling, and vascularization.

**Fig. 3.**
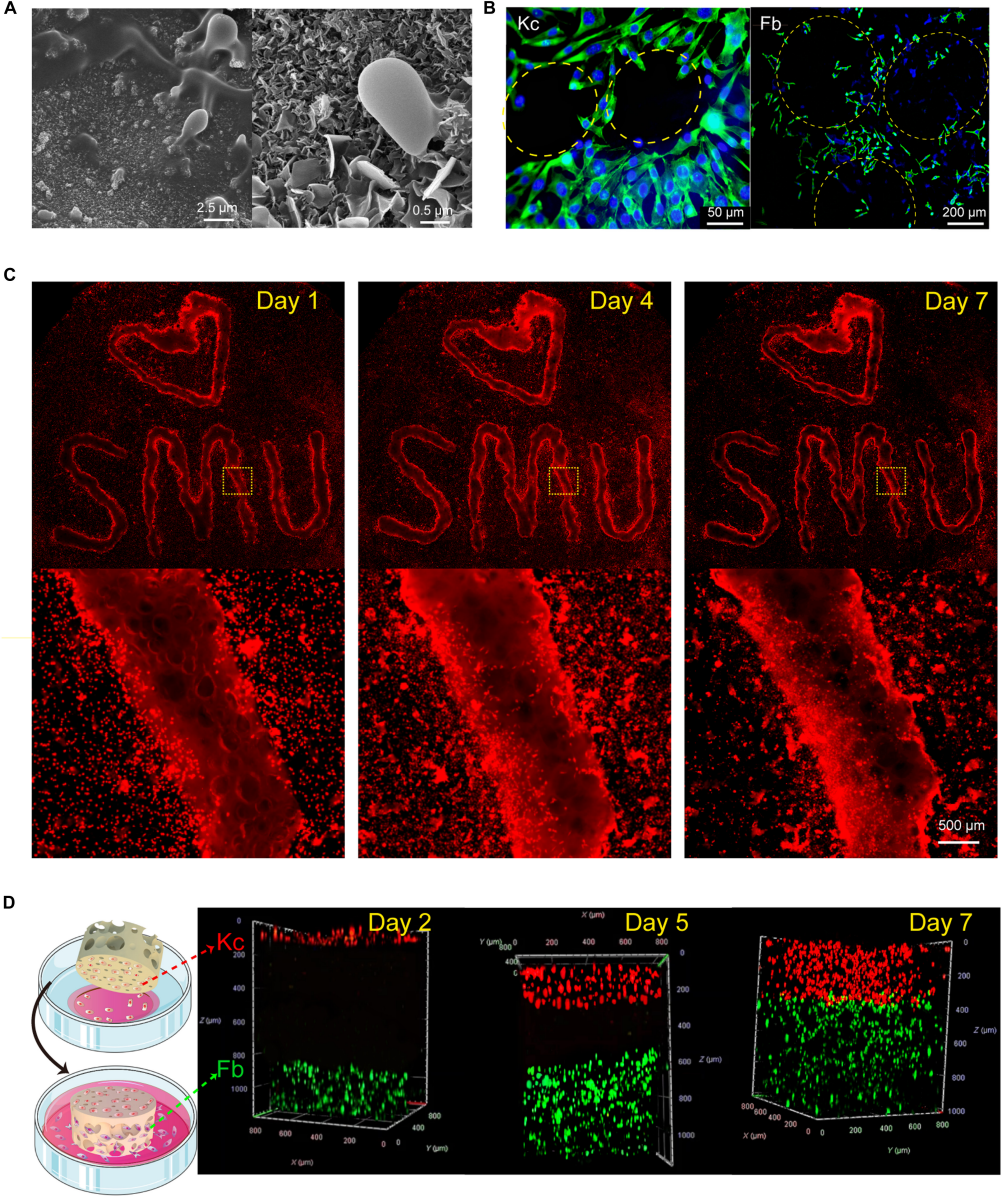
Directional migration and vertical stack of cells on EWS. (A) SEM pictures and (B) cytoskeleton staining of cells on EWS. (C) EWM hydrogels were printed into SMU (Southern Medical University), and the fluorescent cells migrated to the interior of hydrogels with time. (D) Schematic plot of coculture system on EWS and confocal images on days 2, 5, and 7.

After coculturing the EWM hydrogel with cells for a specific duration, the cells were fixed on the hydrogel and examined through scanning electron microscopy (Fig. 3A). The elliptical cells, measuring approximately 2.5 μm in length, can be observed on the surface of the hydrogel. These cells extend pseudopods along the skeleton, indicating the migration of cells supporting the EWM hydrogel. Continuing to enlarge the surface microstructure, it was found that the surface of the EW hydrogel is not smooth and flat, but rather rough and chapped. The surface morphology of the macroporous hydrogel made from EW can also partially explain why it promotes cell migration.

After coculturing EWM with cells for 3 d, the cell cytoskeleton was stained with fluorescein isothiocyanate (FITC)–phalloidin (Fig. 3B). The cells were distributed throughout the hydrogel pores. The fibrous network of skin cells’ cytoskeleton was intact, and the cells were polygonal or spindle-shaped, indicating that the cell morphology cultured on the hydrogel was normal and their function was optimal. In order to visualize the process of cell migration onto the hydrogel, preprinted patterned hydrogels were placed in a culture dish, sterilized, and soaked. A suspension of Fb cells labeled with red fluorescent DiI is seeded onto the hydrogel. Fluorescent images are taken on days 1, 4, and 7 (Fig. 3C). It can be observed that on the first day, the red fluorescence accumulates at the edge of the hydrogel due to the interception effect, resulting in a bright edge around the hydrogel pattern. With the extension of coculture time, the surrounding cells gradually migrate toward the hydrogel. It is evident that the cell density surrounding the hydrogel decreases, while the number of cells on the hydrogel increases and the distribution range expands. This phenomenon further illustrates the ability of EWM to induce cell migration toward the hydrogel. This specific directed migration is intrinsically associated with the EW. When circular EWM hydrogels and GelMA hydrogels were cocultured with fluorescence-labeled cells within a single dish, a distinct cell-excluded zone was observed surrounding the GelMA construct, indicating that peripheral cells do not spontaneously migrate into the GelMA interior (Fig. S4D).

To determine the proper cell density and culture time on EWS, a 3D migration test was conducted (Fig. 3D). 3D imaging demonstrated that on the 5th day, 2 cell types approximately covered the designed layer. However, on the 10th day, the boundary between the 2 cells got blurred, indicating overgrowth and invasion of cells if prolonging culture time. As a result, a 1-d culture of Kc followed by 4-d culture of Fb was adopted as the coculture scheme on EWS.

### EWS accelerated wound healing

The superior properties of EWS in in vitro culture have been well investigated. We next evaluated the efficiency of EWS on full-thickness skin defect models. The mice were divided into 3 groups: CON (untreated wounds), EWM (EWM hydrogels), and EWS (EWS hydrogel with Fb and Kc cells). To ensure sequential wound observation, individual mice were identified by applying gentian violet dye via a skin marker according to the study of Klabukov et al. [41].

**Fig. 4.**
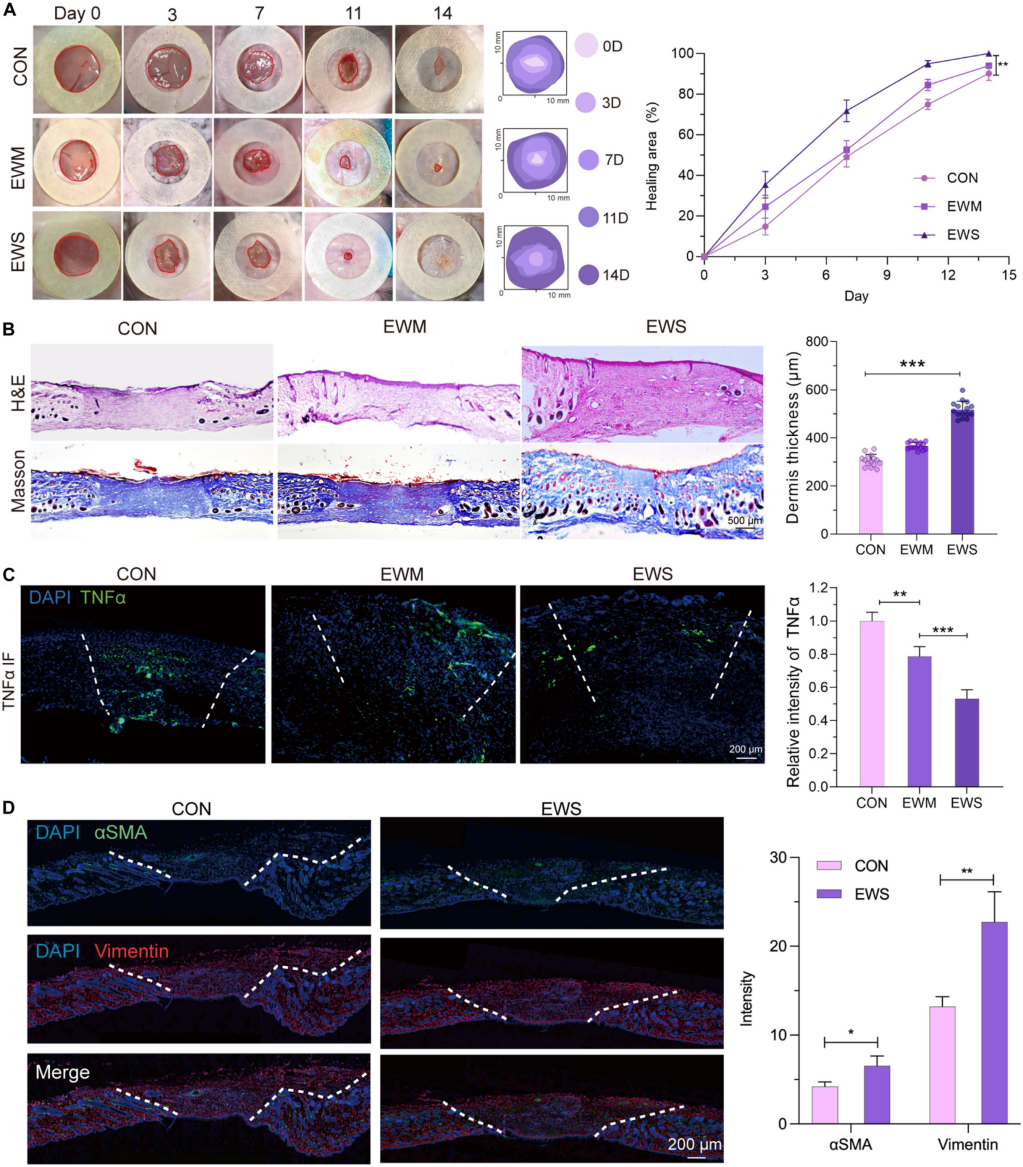
EWS accelerated wound healing of mice. (A) Representative macroscopic wound pictures of each group over 14 d and quantification of wound closure (*n* = 8). (B) Histological staining of skin samples (*n* = 8). (C) Immunofluorescence (IF) and quantification of TNFα (*n* = 8). (D) Immunofluorescence and quantification of healing markers αSMA and vimentin (*n* = 8). *P < 0.05, **P < 0.01, ***P < 0.001.

The gross appearances of wounds for all groups for different time intervals are shown in Fig. 4A. The ratio of the remaining wound area versus the primary area was used as a parameter to check the healing efficiency. Fourteen days after surgery, 90% of the wound was unclosed in the animals of the untreated group, while in the EWM group, the percentage was 94%. However, the EWS group already had 95% closure on the 11th day and complete healing at the designed endpoint.

Further dissection of the reconstructive effect of EWS was obtained by analyzing the histological sections of the regenerative tissues. According to the wound healing stage theory, inflammation lasts for 3 to 7 d and the tissue enters the remodeling phase. Thereby, mice were sacrificed on days 7 and 14 after injury for dynamic observation of the inflammation, re-epithelialization, and ECM reorganization conditions. It was noteworthy that, unlike the other groups, protruding and stacked corneum progressed toward the wound center to refill the defect, and the EWS group demonstrated in situ regeneration of epithelium depending on the hydrogel and cell seeds (Fig. 4B). On the 14th day, wounds within the EWM and EWS groups showed almost complete re-epithelization, while the epidermis thickness of the EWS group was significantly larger and closer to the normal murine skin. The Masson staining also revealed a better arrangement of collagen fiber and thicker regenerated dermis. Abundant neogenesis of hair follicles in the reticular dermis indicated the formation of more complete and functional skin. Organized and directed reconstruction of epidermis and dermis prevented the unordered and pathologic tissue formation and confirmed the feasibility of the bilayer macroporous hydrogel in skin regeneration. The intensity of inflammation marker TNFα of the hydrogel and EWS groups was significantly lower than that of the control on day 7 (Fig. 4C). In the 14th-day samples of EWS, significantly higher levels of αSMA and vimentin were detected when compared with the control (Fig. 4D), which underscored the activated functions of 2 loaded cells.

It was supposed that EWS would not cause an extra inflammatory reaction when served as wound dressings. The in vivo degradation test showed that more than 55% of the EWS hydrogel mass was retained at the end point of the experiment (Fig. S5A). The hydrogel samples did not cause pathological inflammatory responses in the body (Fig. S5B). The mechanical properties and morphology will not change at the acute wound stage (Fig. S5C).

### The effects of EWM validated by RNA sequencing

**Fig. 5.**
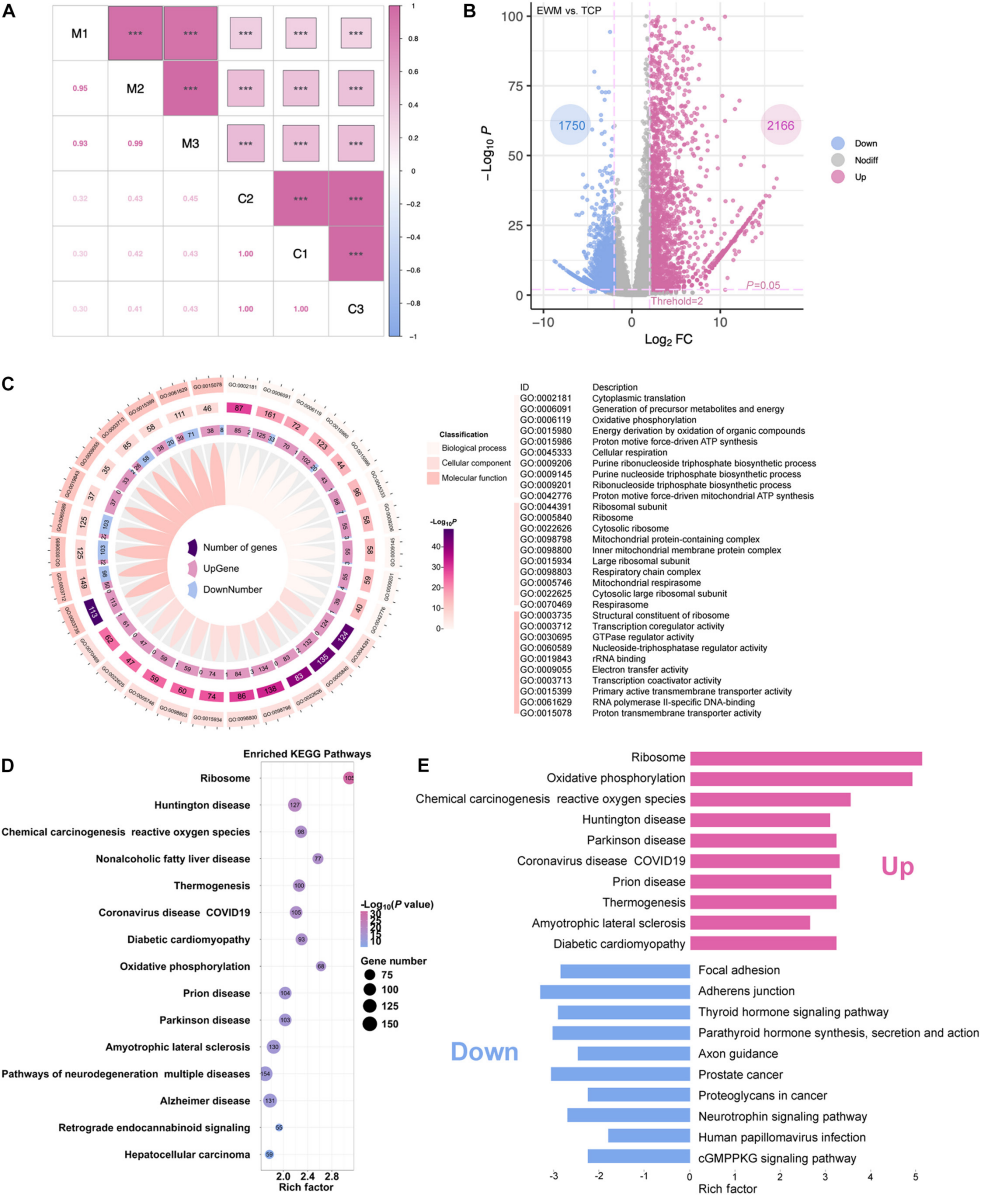
Differential gene analysis and enrichment analysis of EWM-cultured cells. (A) Correlation analysis between different groups. (B) Volcano plot of gene expression. (C) GO functional enrichment analysis. (D) KEGG pathway enrichment analysis. (E) Enrichment of up-regulated and down-regulated pathways.

Gene expression exhibits biological variability among different individuals or populations. In order to mitigate the effects of this variability, it is necessary to identify the correlation of gene expression levels among samples within a group. The correlation coefficients within the EWM-cultured Fb (M1, M2, M3) and the control group cultured on the plate (C1, C2, C3) are all greater than 0.93, while the correlation coefficients between the 2 groups are less than 0.50 (Fig. 5A). This indicates good repeatability within the 2 groups and a significant difference between the groups.

Differential analysis of gene expression in Fb cultured in EWM was performed, and a total of 3,916 DEGs were obtained (Fig. 5B). Figure 5C illustrates the top 10 GO terms that are highly enriched in biological processes, cellular components, and molecular functions. Overall, the transcriptome analysis of cells cultured in EWM reveals a significant enrichment of genes associated with various biological processes, including aerobic respiration capacity, ribosomal components, actin binding, immune system-positive regulation, and protein transcription and translation. Subsequently, the annotation of DEGs will be conducted using the KEGG database in order to examine the impact of EWM culture on cellular pathways (Fig. 5D).

Performing KEGG analysis on the up-regulated and down-regulated genes separately is essential to identify the pathways that are most significantly affected (Fig. 5E). The most highly enriched pathways in this group include ribosome, oxidative phosphorylation, thermogenesis, chemical carcinogenesis, neurodegenerative diseases, diabetic cardiomyopathy, non-alcoholic fatty liver disease, and glutathione metabolism. In the group exhibiting down-regulation, the pathways that showed the highest enrichment include adhesion, hormone-related diseases, cancer-related pathways, neural transmission-related pathways, inflammation-related neutrophils, cyclic GMP-dependent protein kinase G (cGMP-PKG), epidermal growth factor receptor (EGFR), and insulin signaling. The findings suggest that cells cultured in EWM exhibit enhanced energy metabolism and protein synthesis. Combined with previous experimental findings, the present study suggests that the use of EWM can potentially stimulate cell proliferation and migration by increasing mitochondrial production and influencing cell cycle-related activities. The observed down-regulation of cell adhesion indicates that EWM has the potential to decrease cell adhesion and facilitate cell migration toward hydrogels.

**Fig. 6.**
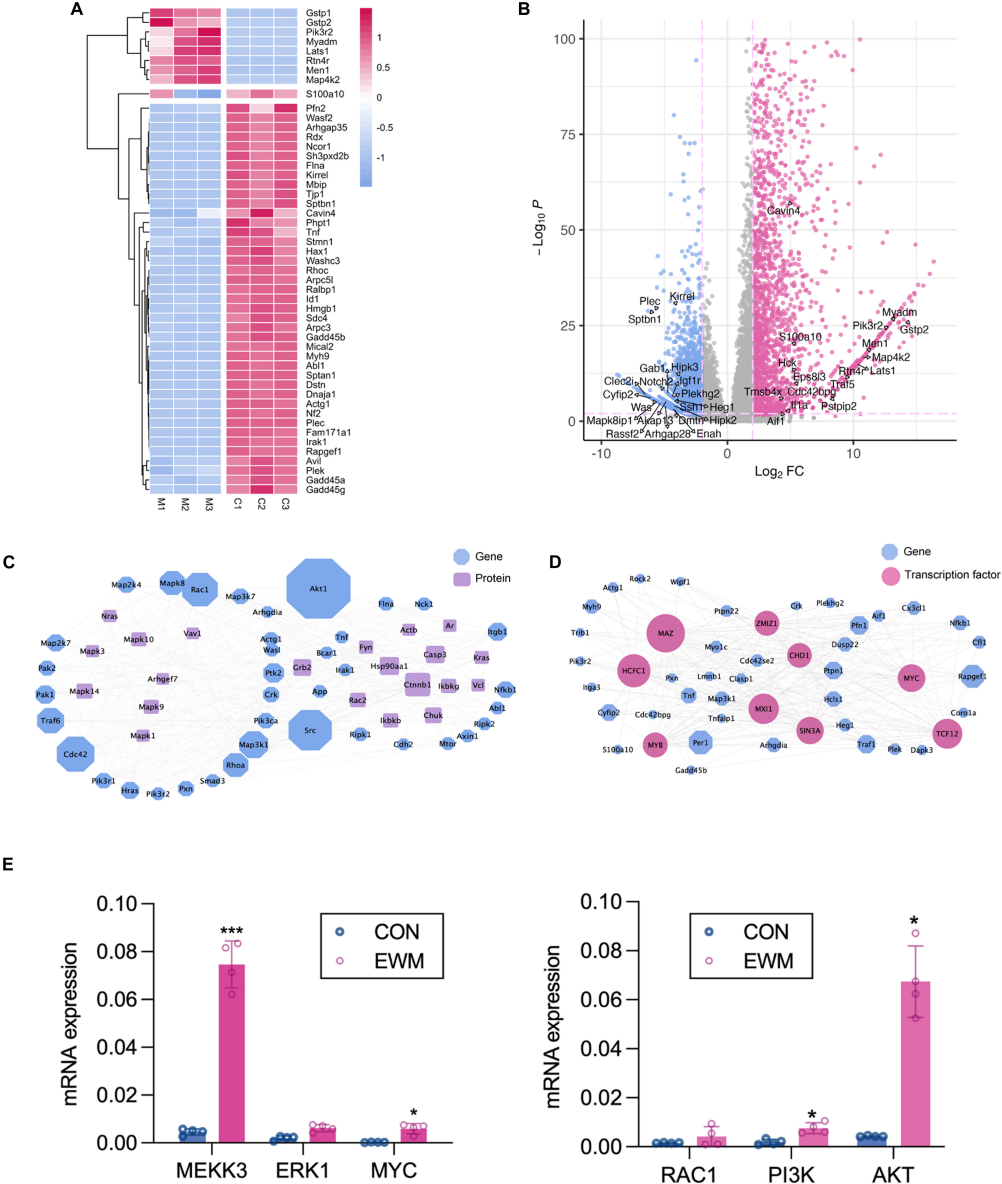
Analysis of migration-related genes. (A) Heatmap of migration-related genes expression. (B) Volcano plot of differential genes with top 20 genes. (C) Net plot of gene transcription factor interaction. (D) Core transcription factor–gene interaction network. (E) RNA expression of MAPK/ERK and PI3K/AKT pathways.

To gain a deeper understanding of the mechanism of EWM enhancing cell migration, we identified genes that were differentially expressed and associated with migration function based on GO annotations. We conducted an analysis on the top 150 genes exhibiting the highest FC. Figure 6A depicts the relative expression levels and clustering outcomes of the migration genes. Among the genes that have shown increased expression, Men1 is a tumor suppressor gene that is associated with the development of multiple endocrine neoplasia syndrome [42]. Myadm, a differentiation marker related with bone marrow, is a negative regulator of cell adhesion and protein kinase C signaling [43]. Among the down-regulated genes, Kirrel is an immunoglobulin superfamily cell adhesion molecule that plays a role in the activation of the Hippo tumor suppressor pathway and the inhibition of cell growth [44].This connection has significant implications for cell shape and the arrangement of transmembrane proteins. The blue polygons depict the fundamental genes within the interaction network, while the purple squares symbolize the core proteins.

The size of the squares in the network connections represents the significance of the genes or proteins (Fig. 6C). Identifying core transcription factors can provide valuable insights into the fundamental regulatory mechanisms. By conducting a comparative analysis with the ENCODE chromatin immunoprecipitation sequencing database, upstream transcription factor regulatory networks were drawn (Fig. 6D). In the protein regulatory network, mitogen-activated protein kinase Mapk, Ctnnb1, heat shock protein Hsp90aa1, and nuclear factor κB (NFκB) inhibitor chuk illustrate the involvement of transcription factors HCFC1, MAZ, MYC, MIX1, and TCF12 in the transcriptional regulation of genes associated with migration.

The aforementioned analysis findings indicate that 2 pathways, MAPK/extracellular signal-regulated kinase (ERK) pathway and phosphatidylinositol 3-kinase (PI3K)/AKT pathway, potentially play a role in enhancing cell proliferation and migration in EWM. The PI3K/AKT pathway is a prominent cellular signaling pathway that holds significant importance in fundamental intracellular processes. It governs various cellular functions such as cell proliferation, growth, cell size, metabolism, and motility [45]. The MAPK/ERK pathway serves as a central signaling hub that integrates various stimuli, such as internal metabolic stress, DNA damage pathways, and changes in protein concentrations [46]. The qPCR test was performed, revealing an increase in the expression levels of these 2 pathways in Fb cultured in EWM (Fig. 6E).

### EWS improves diabetic wound healing

**Fig. 7.**
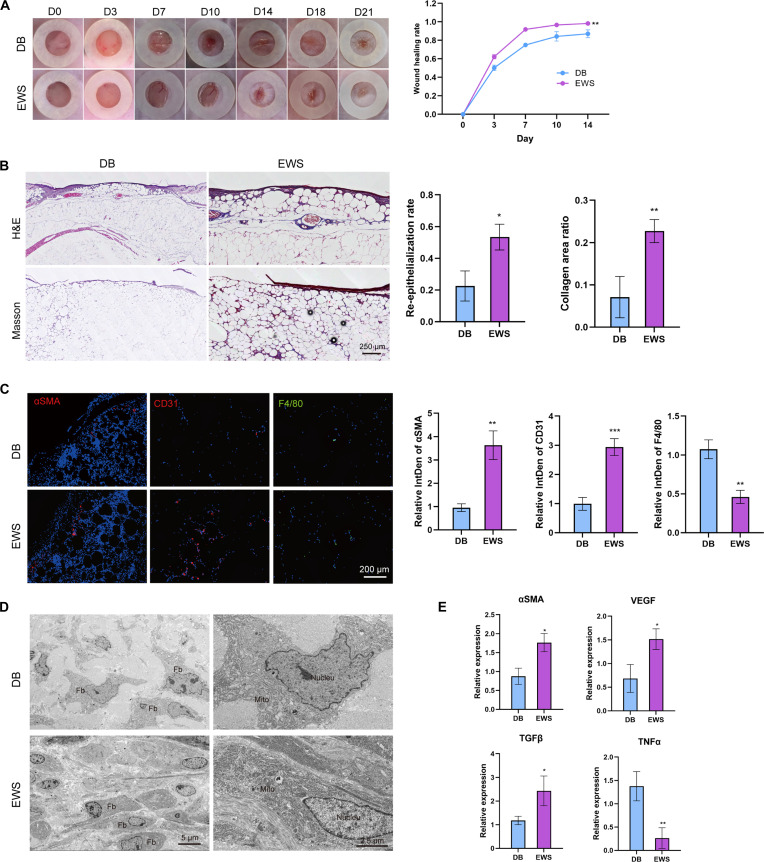
EWS promotes diabetic wound healing. (A) Representative macroscopic photos of diabetic mice over 21 d and the quantification of wound healing rate (*n* = 8). (B) H&E and Masson staining of wound samples at the 7th day, and the quantification of re-epithelialization and collagen area (*n* = 8). (C) Immunofluorescence and quantification of healing markers αSMA, CD31, and F4/80. (D) TEM of regenerative dermis Fb (*n* = 8). (E) qPCR of the 7th wound samples (*n* = 8). *P < 0.05, **P < 0.01, ***P < 0.001.

Impaired cellular migration represents one of the hallmark pathological features of diabetic wound healing, so we applied EWS in diabetic wounds. Dynamic monitoring of the wound healing process revealed remarkable therapeutic advantages in the EWM group, characterized by accelerated wound closure (Fig. 7A). Histopathological analyses further corroborated these findings: H&E staining demonstrated significantly improved epithelialization, while Masson’s trichrome staining revealed abundant deposition of newly synthesized blue collagen fibers, indicating active ECM remodeling (Fig. 7B). At the molecular level, immunofluorescence staining unveiled enhanced ECM regeneration in EWS-treated wounds, as evidenced by increased αSMA-positive cells, and markedly reduced inflammatory cell infiltration (Fig. 7C). These observations were further validated by PCR analysis, which showed significant up-regulation of multiple molecular markers associated with wound healing, providing mechanistic insights into EWS-mediated diabetic wound repair (Fig. 7E).

To gain deeper insights into the regulatory effects of EWS on wound microenvironment, we performed transmission electron microscopy (TEM) to examine Fb ultrastructure. As illustrated in Fig. 7D, EWS-treated wounds exhibited a significant increase in dermal Fb displaying characteristic activated phenotypes: elongated cell bodies, cytoplasm rich in rough endoplasmic reticulum and Golgi apparatus, and particularly notable mitochondrial proliferation. These ultrastructural features collectively indicate enhanced metabolic activity and migratory capacity. The multi-level, comprehensive experimental evidence demonstrates that EWS significantly improves Fb migration within the diabetic wound microenvironment, effectively promoting endothelial cell remodeling and angiogenesis, ultimately leading to accelerated and high-quality diabetic wound healing. These findings provide novel insights for clinical management of diabetic wounds.

## Conclusion

Current clinical mainstays, including collagen-based films, alginate dressings, synthetic hydrogels, and polymeric scaffolds, exhibit inherent trade-offs between biocompatibility, exudate management, mechanical stability, bioactive functionality, and fabrication scalability. The development of EWS represents a significant advancement in biomimetic wound dressings, offering multifaceted advantages for skin regeneration. EWS leverages the innate bioactivity and cost-effectiveness of EW proteins, which are rich in bioactive components such as lysozyme and ovotransferrin. These components confer intrinsic antibacterial properties and promote angiogenesis and cell migration without requiring exogenous growth factors. Notably, EWS demonstrated remarkable efficacy in diabetic wound healing, resolving impaired cellular migration and inflammation while promoting Fb activation and ECM synthesis. These attributes underscore its clinical potential as a low-cost, biocompatible, and functionally adaptive dressing for chronic wounds.

Despite these advancements, this study has limitations. The in vivo experiments were conducted exclusively on murine models, which may not fully replicate human wound healing dynamics. Long-term safety and biodegradability of EWS remain unvalidated, particularly regarding potential immune responses to residual EW antigens. Additionally, the mechanical stability of EWS under dynamic physiological conditions (e.g., shear stress and moisture fluctuations) was not thoroughly tested, which could affect its durability in clinical settings. Compositional analysis of material components constitutes a critical factor in EWS gelation kinetics. Future investigations would benefit from employing chromatographic techniques (e.g., liquid chromatography–mass spectrometry) for precise macromolecular weight distribution profiling. Scalability and sterilization protocols for large-scale production also warrant further investigation.

In summary, EWS emerges as a promising biomimetic hydrogel for skin regeneration, combining hierarchical architecture, inherent bioactivity, and cost-effectiveness. Its ability to mimic natural skin layers, enhance cell communication, and accelerate diabetic wound healing highlights its translational potential. Future research should focus on optimizing EWS for human applications, including clinical trials to validate efficacy and safety, long-term biocompatibility studies, and exploration of its utility in complex wounds (e.g., burns and infected ulcers). Integrating smart functionalities (e.g., stimuli-responsive drug release) or combining EWS with stem cells could further enhance its regenerative capacity. Addressing these challenges will pave the way for EWS to become a mainstream therapeutic option in regenerative medicine.

## Data Availability

Data of this article can be obtained from the corresponding authors on request.
