# Supplementary material for: Hierarchical Porous Egg White Hydrogel Promotes Diabetic Wound Closure through Topography-Guided Cell Recruitment
Source: Biomater Res. 2026 Feb 11;30:0279. doi: 10.34133/bmr.0279 (PMC12891219; doi:10.34133/bmr.0279)
Supplement: Supplementary 1 — Table S1 Figs. S1 to S5 [file bmr.0279.f1.docx]

Table S1 PCR primers

| IL1β | Forward: GCAACTGTTCCTGAACTCAACT  Reverse: ATCTTTTGGGGTCCGTCAACT |
| --- | --- |
| TGFβ1 | Forward: CTCCCGTGGCTTCTAGTGC  Reverse: GCCTTAGTTTGGACAGGATCTG |
| αSMA | Forward: CCCAACTGGGACCACATGG  Reverse: TACATGCGGGGGACATTGAAG |
| bFGF | Forward: TGGTGACCACAAGCTGAATG  Reverse: TCCCTTGATAGACACAACTCCTC |
| FGF7 | Forward: CTCTACAGGTCATGCTTCCACC  Reverse: ACAGAACAGTCTTCTCACCCT |
| EGF | Forward: AGCATCTCTCGGATTGACCCA  Reverse: CCTGTCCCGTTAAGGAAAACTCT |
| VEGF | Forward: GCACATAGAGAGAATGAGCTTCC  Reverse: CTCCGCTCTGAACAAGGCT |
| IL4 | Forward: AGATGGATGTGCCAAACGTCCTCA  Reverse: AATATGCGAAGCACCTTGGAAGCC |
| IL6 | Forward: CCAAGAGGTGAGTGCTTCCC  Reverse: CTGTTGTTCAGACTCTCTCCCT |
| IL13 | Forward: CCTGGCTCTTGCTTGCCTT  Reverse: GGTCTTGTGTGATGTTGCTCA |
| GAPDH | Forward: TCAACAGCAACTCCCACTCTTCCA  Reverse: ACCCTGTTGCTGTAGCCGTATTCA |


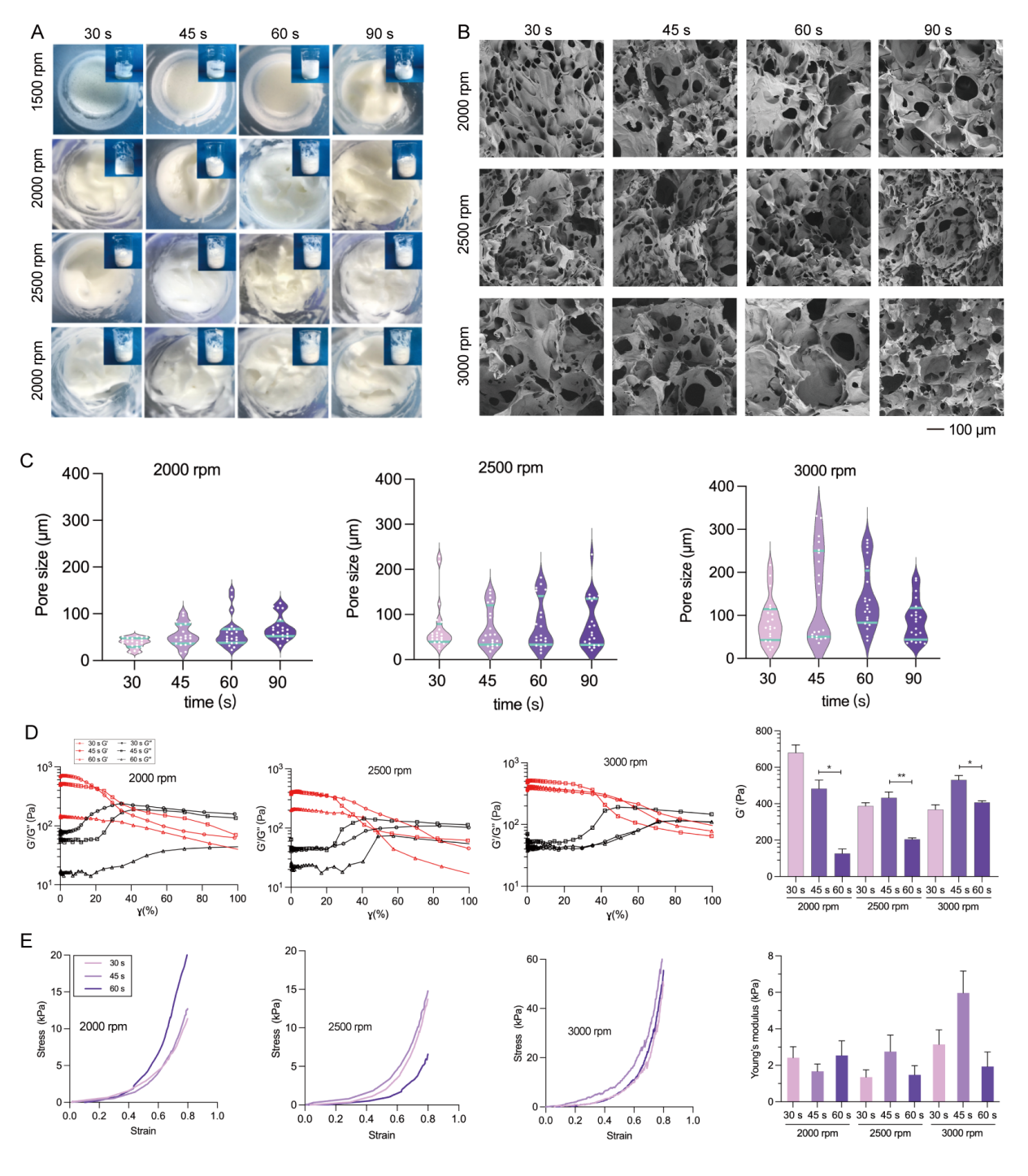


***Figure S1. Influence of different whipping conditions on the pore structure of EWM.***

1. *Gross pictures.*
2. *SEM pictures.*
3. *Pore sizes graphs (n=4).*
4. *Rheology tests and G’ graph (n=6).*
5. *Compression tests and Young’s modulus graphs (n=6).*

*(*: p＜0.05, **: p＜0.01)*


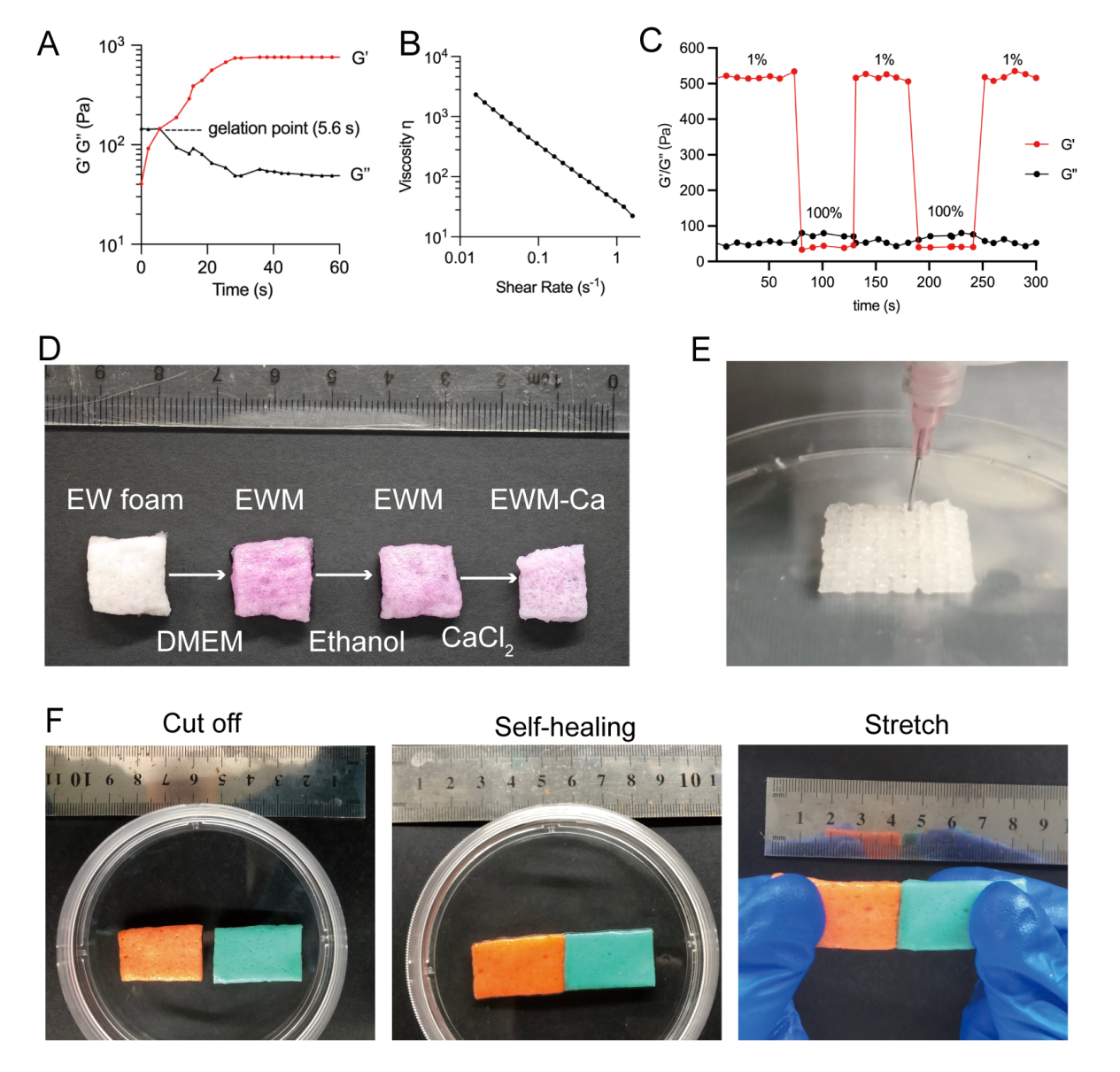


***Figure S2. Self-healing and print-ability of EWM.***

1. *Rheology test during gelation.*
2. *Sheer thinning test.*
3. *Cyclic rheological test with strain switched from 1% to 100%.*
4. *Photos of EWM under different treatments.*
5. *3D print of EWM.*
6. *Self-healing test.*


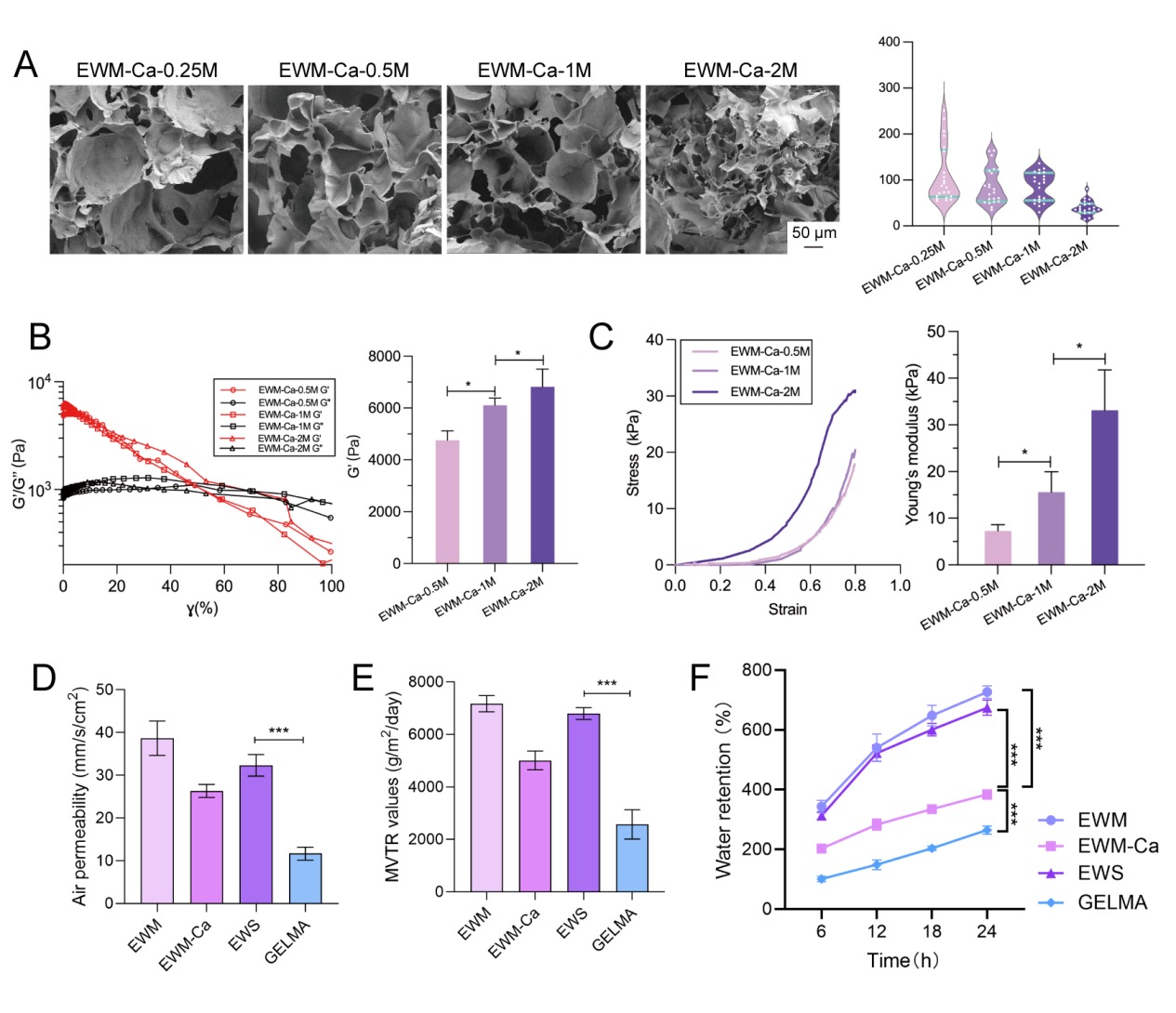


***Figure S3. Secondary crosslinking of EWM and other physical properties.***

1. *SEM pictures and pore sizes plots of EWM-Ca hydrogels made by different concentrations of calcium and the statistics of pores sizes.*
2. *Rheology test and G’ graph.*
3. *Compression test and Young’s modulus graph.*
4. *Air permeability.*
5. *Moisture vapor transmission rate (MVTR).*
6. *Water retention capacity.*

*(*: p＜0.05, **: p＜0.01, ***: p＜0.001)*

*
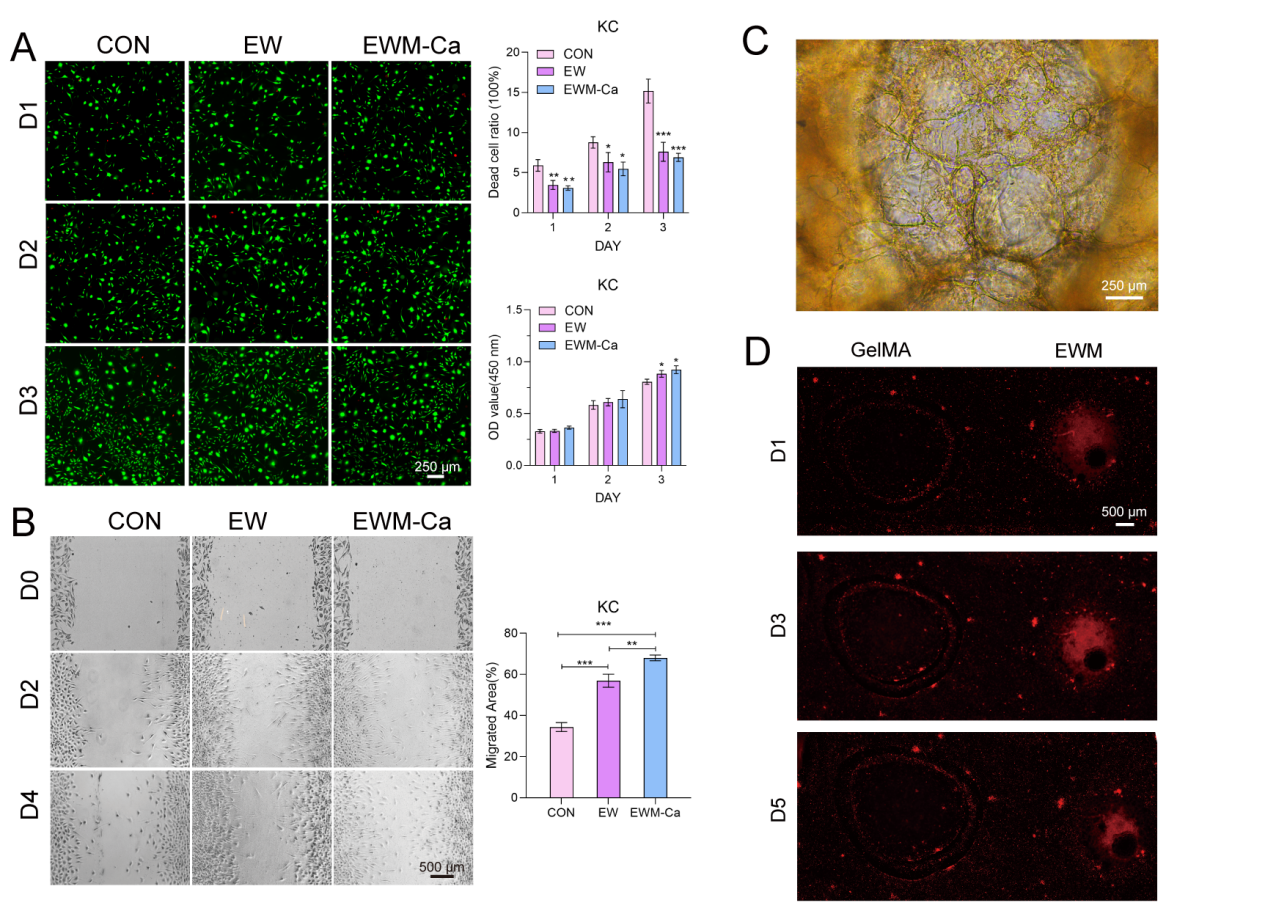
*

***Figure S4. Cell compatibility of EWM-Ca hydrogels to keranoticytes and the migration induction property of EWM hydrogels.***

1. *Live&dead staining and the statistics of dead cell ratio during time.*
2. *Scratch test and the statistics of migrated area.*
3. *Optical microscopy of EWM hydrogels.*
4. *Fluorescent photos of DiI labeled Fb cells cultured in the dish placed with GelMA hydrogels and EWM hydrogels for 5 days.*

*(*: p＜0.05, **: p＜0.01, ***: p＜0.001)*


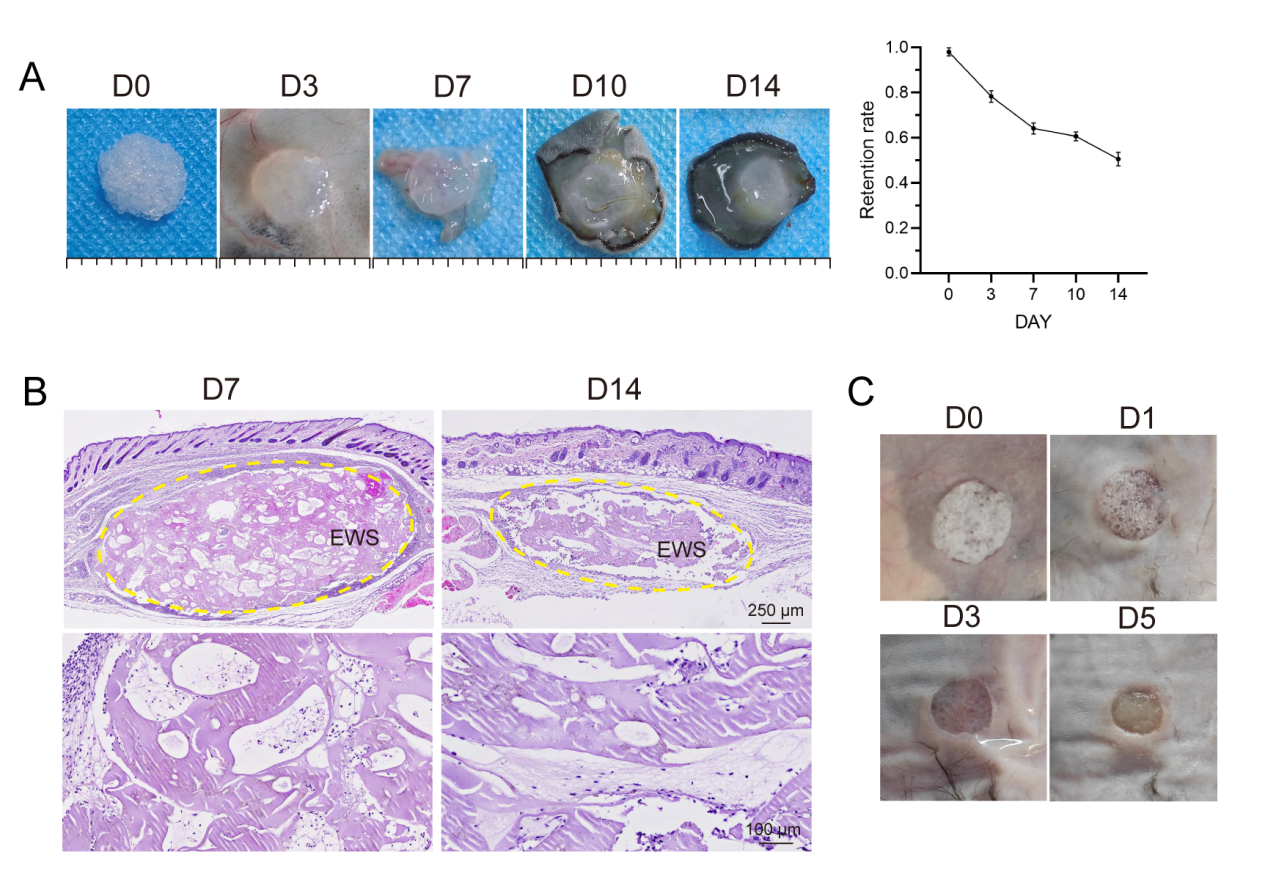


***Figure S5. In-vivo degradation and biocompatibility of EWS.***

1. *Degradation pictures of EWS during 14 days in mice and the retention rate graph.*
2. *HE staining of EWS in vivo. The yellow circles pointed the area of EWS in the tissue slice.*
3. *Morphological Changes of EWS Hydrogel on the Wound Surface.*
